# A decade of genomic history for healthcare-associated *Enterococcus faecium* in the United Kingdom and Ireland

**DOI:** 10.1101/gr.204024.116

**Published:** 2016-10

**Authors:** Kathy E. Raven, Sandra Reuter, Rosy Reynolds, Hayley J. Brodrick, Julie E. Russell, M. Estée Török, Julian Parkhill, Sharon J. Peacock

**Affiliations:** 1 University of Cambridge, Department of Medicine, Addenbrooke's Hospital, Cambridge CB2 0QQ, United Kingdom;; 2 The Wellcome Trust Sanger Institute, Wellcome Genome Campus, Hinxton, Cambridge CB10 1SA, United Kingdom;; 3 British Society for Antimicrobial Chemotherapy, Birmingham B1 3NJ, United Kingdom;; 4 North Bristol NHS Trust, Southmead Hospital, Bristol, BS10 5NB, United Kingdom;; 5 Culture Collections, Public Health England, Porton Down, Salisbury SP4 0JG, United Kingdom;; 6 Public Health England, Clinical Microbiology and Public Health Laboratory, Addenbrooke's Hospital, Cambridge CB2 0QQ, United Kingdom;; 7 Cambridge University Hospitals NHS Foundation Trust, Cambridge CB2 0QQ, United Kingdom;; 8 London School of Hygiene and Tropical Medicine, London, WC1E 7HT, United Kingdom

## Abstract

Vancomycin-resistant *Enterococcus faecium* (VREfm) is an important cause of healthcare-associated infections worldwide. We undertook whole-genome sequencing (WGS) of 495 *E. faecium* bloodstream isolates from 2001–2011 in the United Kingdom and Ireland (UK&I) and 11 *E. faecium* isolates from a reference collection. Comparison between WGS and multilocus sequence typing (MLST) identified major discrepancies for 17% of isolates, with multiple instances of the same sequence type (ST) being located in genetically distant positions in the WGS tree. This confirms that WGS is superior to MLST for evolutionary analyses and is more accurate than current typing methods used during outbreak investigations. *E. faecium* has been categorized as belonging to three clades (Clades A1, hospital-associated; A2, animal-associated; and B, community-associated). Phylogenetic analysis of our isolates replicated the distinction between Clade A (97% of isolates) and Clade B but did not support the subdivision of Clade A into Clade A1 and A2. Phylogeographic analyses revealed that Clade A had been introduced multiple times into each hospital referral network or country, indicating frequent movement of *E. faecium* between regions that rarely share hospital patients. Numerous genetic clusters contained highly related *vanA*-positive and -negative *E. faecium*, which implies that control of vancomycin-resistant enterococci (VRE) in hospitals also requires consideration of vancomycin-susceptible *E. faecium*. Our findings reveal the evolution and dissemination of hospital-associated *E. faecium* in the UK&I and provide evidence for WGS as an instrument for infection control.

*Enterococcus faecium* is a commensal of the gastrointestinal tract (Endtz et al. 1997) and an important cause of healthcare-associated infection, particularly in immunocompromised patients (Montecalvo et al. 1996; Patel 2003). In 2014, enterococci were the second most commonly isolated microorganism from ICU-acquired bloodstream infections in Europe (European Centre for Disease Prevention and Control 2015). In addition to carriage by hospital patients (Endtz et al. 1997), *E. faecium* can also be shed into the environment, where this can persist for extended periods of time (Falk et al. 2000; Neely and Maley 2000). Transmission of *E. faecium* from one patient to another through direct or indirect spread underlies nosocomial infection, the prevention of which depends on infection control interventions, including hand hygiene, environmental cleaning, and effective detection and control of outbreaks.

Bacterial typing is used to add evidence to infection control epidemiology during outbreak investigations and to gain an understanding of the population structure of *E. faecium*. Multilocus sequence typing (MLST) was described for *E. faecium* in 2002 (Homan et al. 2002), which assigns most clinical isolates worldwide to a hospital-associated clonal complex (CC)17 (Willems et al. 2005) based on eBURST. Hospital-associated strains are generally resistant to ampicillin (Willems et al. 2005) and quinolones (Leavis et al. 2006) and are enriched in mobile elements, putative virulence determinants, and resistance to antibiotics (Kim and Marco 2014). eBURST clusters multilocus sequence types (STs) into CCs based on identity of five of the seven MLST loci, but this is less reliable when identifying genetic relatedness in highly recombining species, including *E. faecium* (Turner et al. 2007).

More recently, it has been proposed that CC17 consists of two distinct groups of STs based on Bayesian Analysis of Population Structure (BAPS) analysis of MLST loci (BAPS groups 2-1 [containing ST78] and 3-3 [containing ST17 and ST18]) (Willems et al. 2012). BAPS 3-3 is associated with nosocomial isolates, while BAPS 2-1 is associated with animal isolates and may have a distinct evolutionary history (Willems et al. 2012). The accuracy of MLST has been evaluated using whole-genome sequencing (WGS) in three previous studies. Howden et al. (2013) reported that five of the seven MLST genes in *E. faecium* are under recombination and identified two of 61 isolates with a discrepancy between WGS data and MLST, a finding supported by van Hal et al. (2016), while Pinholt et al. (2015) compared MLST to WGS data for 132 *E. faecium* isolates from across Denmark and found good correlation.

Four WGS-based studies of bacterial collections ranging in size from 61 to 132 *E. faecium* isolates have been published to date (Howden et al. 2013; Lebreton et al. 2013; Pinholt et al. 2015; van Hal et al. 2016). A study of a global collection of 73 *E. faecium* from a range of sources reported the presence of two clades (Clade A1 and B) associated with human clinical and commensal isolates, respectively, together with a third genetically distinct clade associated with animals (Clade A2) (Lebreton et al. 2013). A close genetic relationship was established between *vanB* VRE and vancomycin-susceptible enterococci (VSE) by a study conducted in Australia (Howden et al. 2013). Additionally, WGS studies revealed transmission events within a hospital and suggested that inter- and intra-regional spread of VRE clones may be occurring (Howden et al. 2013; Pinholt et al. 2015; van Hal et al. 2016). Current knowledge gaps include studies that relate to *vanA* VRE (the dominant transposon in the United States and Europe), as well as comprehensive studies of *E. faecium* population dynamics at national and international levels. Here, we use WGS to define the population structure of *E. faecium* clinical isolates in the United Kingdom and Ireland (UK&I) over a decade. These data were further mined to evaluate the robustness of the current MLST typing scheme and to describe the evolution of vancomycin resistance encoded by *vanA* in this setting.

## Results

### Study design and bacterial isolates

We analyzed whole-genome sequence data for 506 *E. faecium* isolates (250 vancomycin-resistant *E. faecium* [VREfm], 256 vancomycin-susceptible *E. faecium* [VSEfm]) drawn from reference (National Collection of Type Cultures [NCTC] *n* = 11) and national (British Society for Antimicrobial Chemotherapy [BSAC] *n* = 495) collections. NCTC isolates consisted of three VREfm and eight VSEfm deposited between 1946 and 2007 and isolated before 1997. The BSAC collection consisted of isolates submitted to a bacteremia resistance surveillance program (www.bsacsurv.org) between 2001 and 2011 by 40 hospitals across the UK&I.

### Comparison of MLST and WGS

We derived the ST from the WGS data. This resolved 53 STs for 501/506 isolates (Supplemental Fig. S1), 96% of which belonged to CC17. The remaining five isolates could not be assigned to an ST because the MLST locus *pstS* had been deleted, together with five contiguous genes (three hypothetical proteins and two insertion sequence [IS] elements) (Supplemental Fig. S2). A homolog of *pstS* (Aus0004_02035) was identified in all study isolates, which was contiguous with a *pst* operon (Supplemental Fig. S2). This suggests that the MLST locus is a homolog of the actual housekeeping gene. Interrogation of the Aus0004 genome with the MLST primers for *pstS* revealed that these bound specifically to the MLST *pstS* locus and so only identified the homolog.

**Figure 1.**
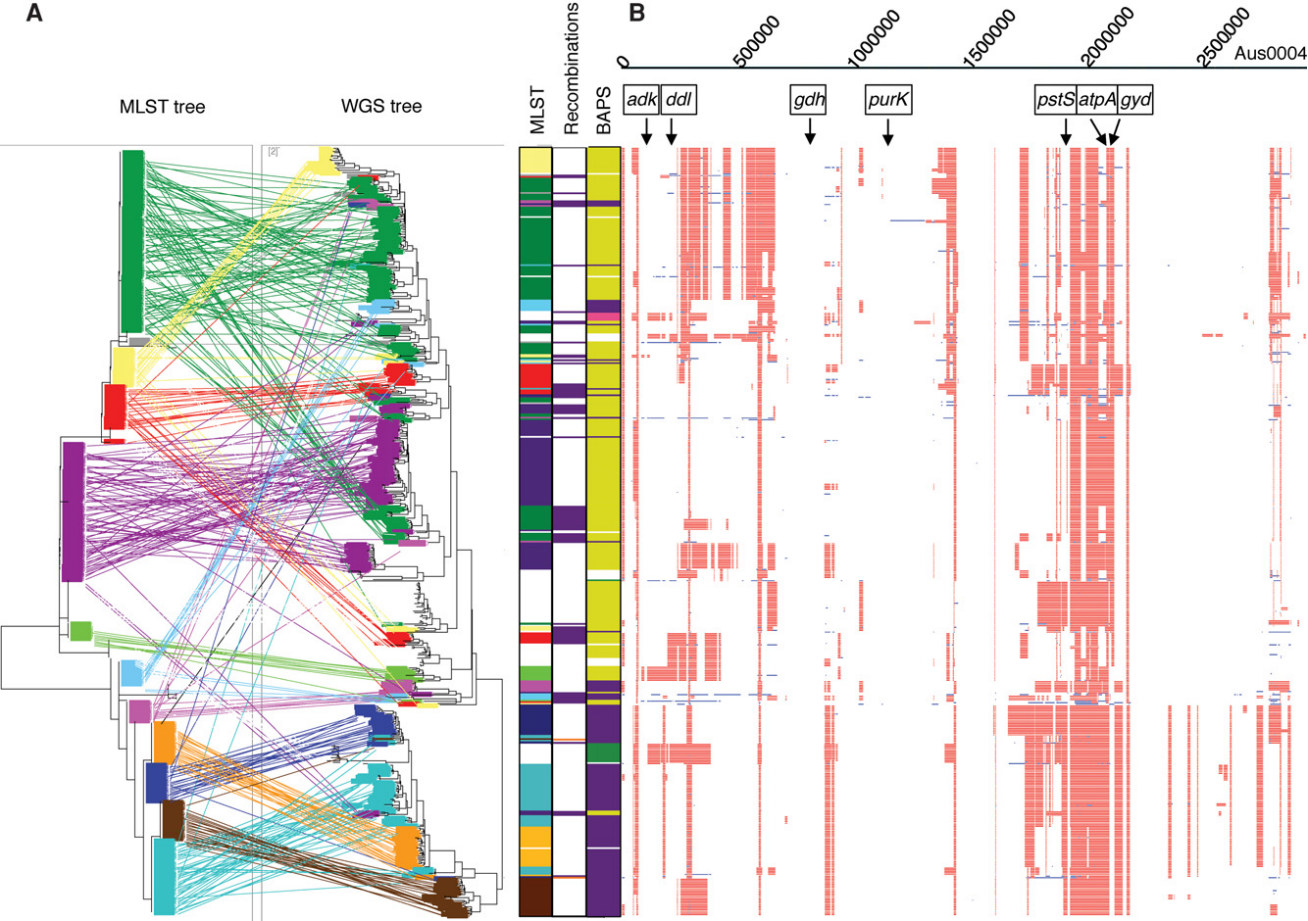
Concordance between MLST and WGS data. (*A*, *left*) Tanglegram linking isolates in phylogenetic trees based on WGS and concatenated MLST loci. A different color is used to represent each ST present in more than one position in the WGS tree. Bars to the *right* show the STs indicated on the tree, the genetic basis for discrepancies between MLST and WGS (recombination = purple; point mutation = orange), and BAPS groups (BAPS 2-1 = purple, BAPS 2-3 = pink, BAPS 3-1 = green, BAPS 3-3 = yellow, unknown = white), with the positions on these bars relating to the position of the isolate on the WGS tree. (*B*) Recombination across the genome. Red indicates a recombination event found in more than one isolate; blue, a recombination event unique to that isolate. MLST genes and reference sequence are indicated at the *top*.

We then undertook a comparative phylogenetic analysis of concatenated MLST loci versus WGS for a subset of 477 VREfm and VSEfm isolates that resided in a closely related genetic cluster (Clade A; see below). This revealed numerous discrepancies between the two methods with a conservative estimate of 80/477 (16.7%) isolates lacking congruence and with dispersal of isolates belonging to the same ST throughout the WGS tree (Fig. 1). Four MLST loci (*atpA*, *gyd*, *pstS*, *ddl*) were located in recombination hotspots and contributed to 78/80 of these discrepancies. In contrast, the remaining three MLST loci showed very limited diversity. Despite the size of the collection, we identified just three, two, and two alleles, respectively, for *adk*, *gdh*, and *purK*. Furthermore, a single allele accounted for 99.2%, 99.8%, and 93.7% of the 477 isolates, respectively, demonstrating that these three loci contributed very little to differentiation between isolates. We investigated the performance of BAPS, which has been proposed as a replacement for clustering by MLST CC. Six out of the 13 BAPs groups described by Willems et al. (2012) were identified based on 32 STs (473/506 isolates) in our collection, with BAPS groups 3-3 (62% of isolates) and 2-1 (31%) being the most common (Supplemental Fig. S1). Annotation of the WGS tree with BAPS groups showed two distinct clusters predominantly containing BAPS 3-3 and 2-1, respectively, but multiple discrepancies between BAPS and WGS were also observed.

### UK&I *E. faecium* genomes in a global genetic context

**Figure 2.**
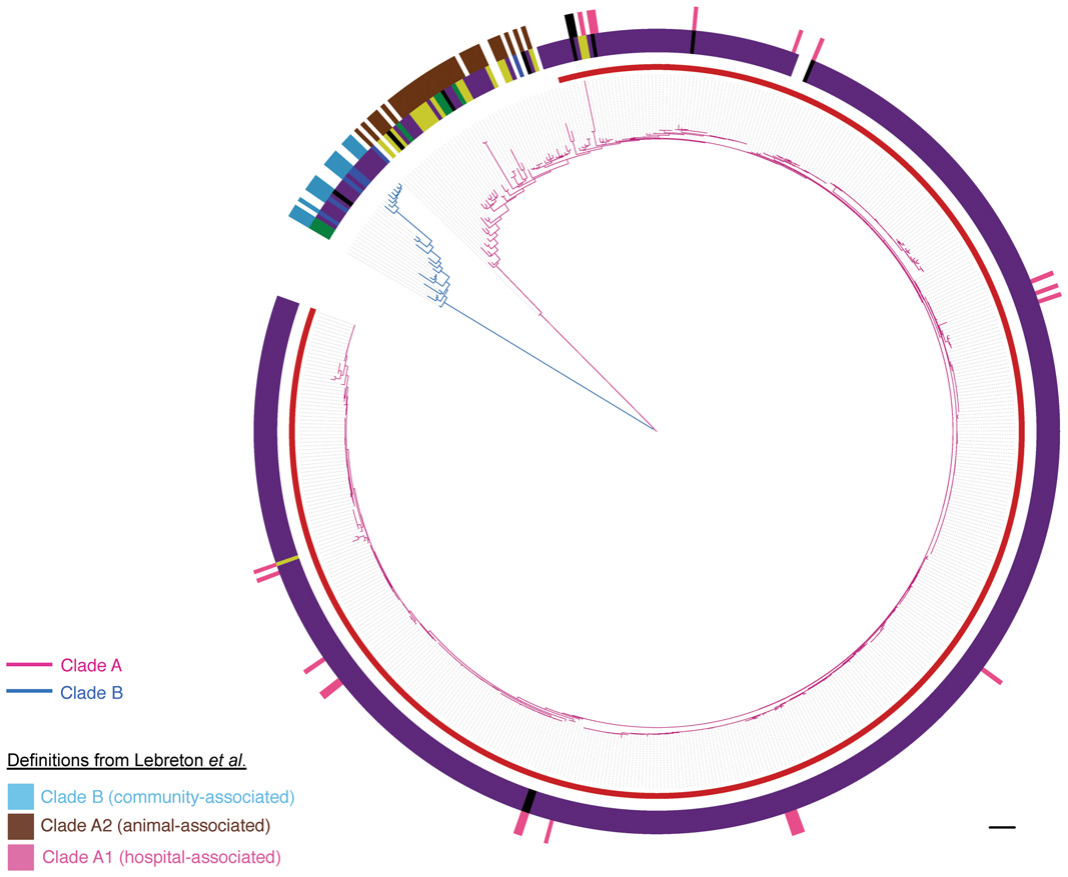
Population structure of *E. faecium*. Maximum likelihood tree based on SNPs in the 1288 genes core to the 506 isolates from this study and 73 isolates reported by Lebreton et al. (2013). Pink branches indicate Clade A; blue branches, Clade B. Inner incomplete red ring denotes the clonal expansion of Clade A. Middle ring shows the isolate source (yellow, animal; purple, clinical; blue, nonhospital; black, hospital-associated feces/surveillance/outbreak/unknown; green, other; white, unknown). Outer ring shows isolates from Lebreton et al. (2013) colored by the clade to which they were originally assigned (pink, Clade A1; brown, Clade A2; blue, Clade B; black, hybrid Clade A1/B). Scale bar, 9593 SNPs.

We combined WGS data for the 506 study isolates with WGS data for 73 isolates described in a study by Lebreton et al. (2013), in which a clade structure was identified consisting of Clades A1 (hospital-associated), A2 (animal-associated), and B (community-associated). The comparison was based on genes conserved across the two collections (*n* = 1288), from which single-nucleotide polymorphisms (SNPs) were identified and used to construct a maximum likelihood tree. This confirmed previous observations that the *E. faecium* population splits into two major lineages (Clades A and B) but did not support the subdivision of Clade A into Clades A1 and A2 (Fig. 2). The Lebreton Clade A2 isolates were basal (ancestral) to Clade A1, and the phylogeny was consistent with a rapid clonal expansion of Clade A from a single progenitor (Fig. 2). We sought a methodological explanation for this difference. In our study we defined core genes as being present in 99% of isolates and Lebreton et al. (2013) used a definition of 100% of isolates, but a reanalysis based on 100% showed that the phylogeny shown in Figure 2 was replicated (Supplemental Fig. S3). Furthermore, it was possible to accurately reproduce the phylogeny created by Lebreton et al. (2013) for the 73 isolates alone (Supplemental Fig. S3). This indicates that our findings relate to the isolate collection (including its larger numerical size) as opposed to differences in analysis.

The majority (97%) of the BSAC collection resided in the clonal expansion of Clade A, together with all three NCTC VREfm isolates, including two from the first clinical case reports of vancomycin-resistant *E. faecium* in 1988 (Uttley et al. 1988). Nine study isolates from geographically dispersed locations (London *n* = 2, East of England *n* = 1, West Midlands *n* = 1, South-West *n* = 1, Northern Ireland *n* = 1, Ireland *n* = 1, Scotland *n* = 1, NCTC *n* = 1) resided in Clade B. Overall, the clonal expansion of Clade A had considerably lower genetic diversity (median 2174 SNPs, range 0–5701 SNPs, interquartile range (IQR) 1538–2772 SNPs) than either Clade B (median 11,673 SNPs, range 2957–18,135, IQR 8289–14,627 SNPs) or the isolates that were basal to Clade A (median 6833 SNPs, range 20–14,206 SNPS, IQR 4682–8098 SNPs).

### Phylogeography of *E. faecium* in the UK&I

**Figure 3.**
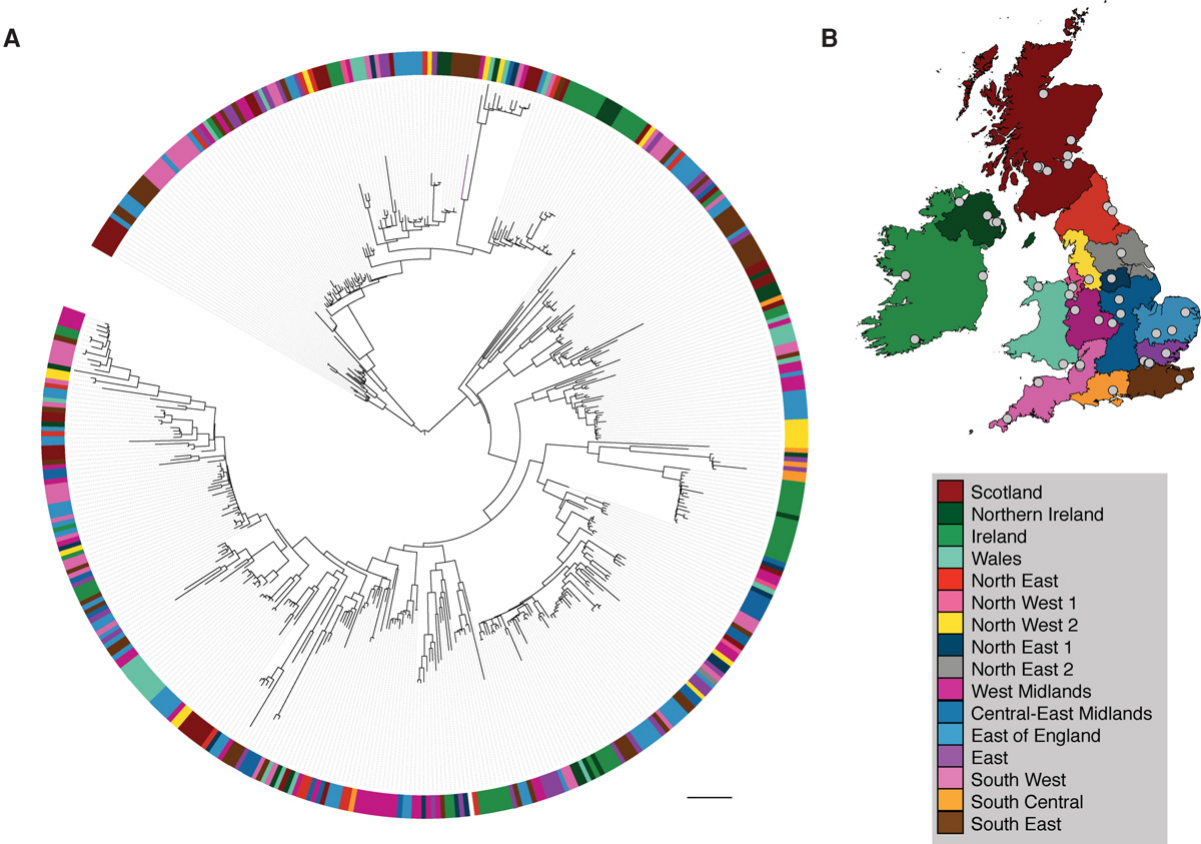
Geographic distribution of *E. faecium* lineages across the UK&I. (*A*) Maximum likelihood tree based on SNPs in the core genome for national isolates belonging to the clonal expansion of Clade A. Colors shown in the colored circle represent the referral network or country where the isolates were cultured. Scale bar, 90 SNPs. (*B*) Map of regions and referral networks described previously (Donker et al. 2012). Centers that submitted samples to the BSAC between 2001 and 2011 are represented by gray dots. Reproduced from Reuter et al. (2016).

Our sampling strategy provided the opportunity to describe the phylogeny of *E. faecium* over time and place in the UK&I. A phylogenetic tree based on 8808 SNPs in the core genome of the Aus0004 reference and 477 study isolates contained in the clonal expansion of Clade A was analyzed by hospital referral network for England (Donker et al. 2012), and by country for Wales, Scotland, Northern Ireland, and Ireland (Fig. 3). We found widespread dissemination of Clade A and a pattern consistent with multiple introductions into each referral network or country. Superimposed on this dispersed pattern were small clusters within the referral networks and countries, which are likely to represent localized transmission and clonal expansion. We also identified two clusters that were geographically restricted to Ireland/Northern Ireland (17 isolates from 2007–2011 and 18 isolates from 2004–2010). Evidence for connectivity and transmission between different hospitals both within a referral network/country and in different referral networks/countries was identified, including nine isolate pairs from different hospitals in the same referral network/country and eight isolate pairs from different hospitals in different referral networks/countries, each pairing being within zero to six core genome SNPs of each other. Analysis by individual hospitals replicated the findings for referral networks, with dispersal of isolates throughout the tree combined with clusters of isolates. Isolates from each year of the study were distributed throughout the tree, although one subpopulation appeared to have emerged after 2005 and consisted of 126/131 isolates originating between 2006 and 2011 (Supplemental Fig. S4).

### Genetic analysis of vancomycin resistance and virulence

**Figure 4.**
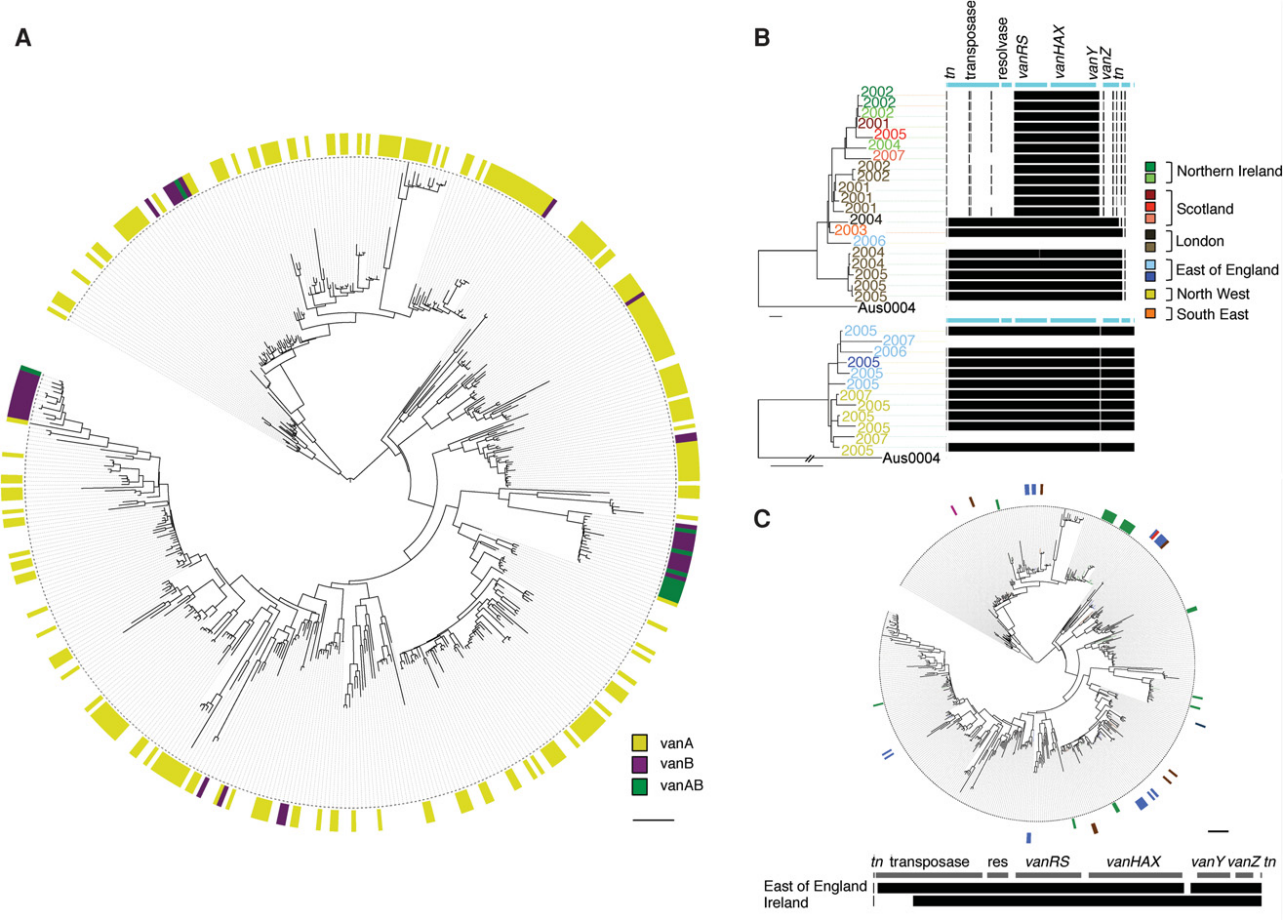
Vancomycin resistance in *E. faecium* from the UK&I. (*A*) Relationship between VREfm and VSEfm. Maximum likelihood tree of 477 study isolates belonging to the clonal expansion of Clade A. Colored circle denotes presence of *vanA* (yellow), *vanB* (purple), *vanA* and *vanB* (green), or none (vancomycin susceptible, white). Scale bar, 90 SNPs. (*B*) Acquisition of vancomycin resistance within hospitals. Maximum likelihood trees of two clusters labeled by year of isolation and colored by hospital: genes present (black) or absent (white) from the *vanA* transposon. Scale bar, 27 SNPs. (*C*) Locally prevalent transposons. Maximum likelihood tree of national isolates belonging to the clonal expansion of Clade A. Colored circles denote isolates carrying transposons overrepresented in Ireland (inner) or the East of England (outer) colored by referral network or country (green, Ireland; blue, East of England; brown, London; navy, Yorkshire; pink, West Midlands; red, North East). (*Bottom*) Genetic content of the *vanA* transposon for both transposon types. Scale bar, 90 SNPs.

We then analyzed the genetic basis of vancomycin resistance in 250 VREfm isolates. The majority (215/250; 86%) carried *vanA*, with the remainder carrying *vanB* (*n* = 25) or both genes (*n* = 10). All but one VREfm belonged to the clonal expansion of Clade A. One Clade B isolate was *vanA*-positive, demonstrating that vancomycin resistance was not restricted to the hospital-associated lineage. Annotation of the tree with *vanA* and *vanB* showed a close relationship between VREfm and VSEfm (Fig. 4A). There was also a striking difference in distribution of the two elements, with *vanA* distributed throughout the tree and *vanB* predominantly (36/44 isolates) located in four genetic clusters (Fig. 4A). Mapping sequence data to reference transposons revealed multiple small- and large-scale variations in genetic content in the *vanA* transposon, but limited genetic variation in the *vanB* transposon (zero to five SNPs difference within each cluster) (Supplemental Fig. S5). BLAST analysis of the transposon insertion sites revealed that *vanA* was predominantly located on plasmids (161 of the 162 that could be identified: pLG1 [*n* = 80], pIP816 [*n* = 45], or comparable matches to pLG1, pF856, p5753cA, and pS177 [*n* = 36]), while 42/44 *vanB* transposons were inserted into the chromosome. Within each cluster, the *vanB* transposons were inserted at identical sites in the chromosome, suggesting acquisition followed by clonal expansion. These results indicate that *vanA* has been repeatedly acquired and lost in the UK&I collection and that *vanB* has been acquired a limited number of times and retained.

Having observed the mobility of *vanA* across the bacterial population, we undertook an analysis of *vanA* within individual referral networks (England) and countries. VREfm clusters predominantly harbored identical *vanA* transposons (Fig. 4B), indicating a single acquisition and subsequent clonal expansion. One transposon was found exclusively in Ireland (21/21), and a second predominantly (26/29) in the East of England (*n* = 19) and London (*n* = 7). These two transposons were located in multiple distinct positions on the phylogenetic tree (seven and five positions, respectively), suggesting that they had been acquired multiple times (Fig. 4C).

Eleven *E. faecium* isolates that were phenotypically susceptible to vancomycin also carried *vanA* (*n* = 1) or *vanB* (*n* = 10). The ten *vanB*-positive VSEfm isolates resided in a single cluster spanning 5 yr and lacked the *vanRB* and *vanSB* regulatory genes, which explains the discrepancy between the phenotype and genotype. A possible explanation for the retention of the *vanB* transposon in the absence of a selective advantage relating to vancomycin resistance is the presence of a secondary fitness advantage arising from other genes carried by the transposon. To investigate this, we analyzed the pan-genome of the 506 isolates to identify genes that were unique to *vanB-*positive isolates. Twenty-nine genes were identified, including a bacteriocin, integrase, and excisionase (Supplemental Table S1). The excisionase suggests that these transposons retained the ability to excise from the genome, while the advantage associated with a bacteriocin may explain its persistence. Of note, the single *vanB* isolate cultured prior to 1997 lacked this bacteriocin.

Finally, we evaluated whether Clades A and B varied in their composition of putative virulence determinants. A comparison of eight *E. faecium* virulence genes from the Virulence Factor Database (VFDB) identified four virulence factors (*ebpA*, *esp*, *hyl*, *scm*) that were present in ≥50% of Clade A isolates but were absent from Clade B (Supplemental Fig. S6), one of which (*esp*) was only identified in the clonal expansion of Clade A (Supplemental Fig. S6).

## Discussion

MLST has been used for over a decade to describe the population structure of *E. faecium* and remains the cornerstone for defining *E. faecium* populations. A striking finding of this study was that the currently used MLST scheme is not robust. Our analyses revealed considerable discrepancy between WGS and MLST and showed that this had resulted from recombination. This contrasts with the study by Pinholt et al. (2015), which identified good correlation between WGS and MLST of 132 *E. faecium* isolates. While discrepancies have been reported previously (Howden et al. 2013; van Hal et al. 2016), our large and comprehensive study extends current knowledge of the problem. We also identified five isolates for which one of the MLST genes had been excised; these bacteria could survive because the gene used in the MLST scheme is a homolog of the phosphate binding protein housekeeping gene. Additionally, we identified discrepancies between WGS and the BAPS groups described by Willems et al. (2012), likely due to the fact that these groupings are based on concatenated MLST genes. The evidence base from this and other studies begins to build the case of need to replace *E. faecium* MLST with WGS, which over time will become increasingly feasible beyond the research setting.

By using WGS, we identified two distinct lineages within *E. faecium* that were consistent with the previously described Clades A and B (Galloway-Peña et al. 2012; Palmer et al. 2012). However, we found no evidence for the split of Clade A into the two discrete subclades (Clades A1 and A2) proposed previously (Lebreton et al. 2013). This difference could be due to the larger collection of isolates used here or may be a feature of the UK&I population. The majority of clinical isolates belonged to a clonal expansion of Clade A, which appears to have emerged once from a progenitor lineage associated with animals. These data provide new insights into the emergence and evolution of the hospital-associated lineages of *E. faecium*.

In this study, the clade described previously as community-associated (Clade B) contained more clinical isolates than community isolates (*n* = 15 and *n* = 8, respectively). This may be expected since the study collection contained predominantly hospital-associated bacteremia isolates. However, similar numbers of hospital and community isolates were identified in Clade B in the study by Lebreton et al. (2013) (*n* = 7 and *n* = 8, respectively), indicating that there is insufficient evidence to describe this clade as community-associated. We propose that the association between clades and origin (hospital/community) be reframed, a process that will gain traction from large-scale national and international studies. This may be aided by a change in nomenclature to Clade 1, Clade 2, etc., as existing and new lineages are identified.

The national distribution of *E. faecium* revealed by WGS in our study indicates that clones frequently move between healthcare networks that more rarely share hospital patients. This finding is supported by studies based on WGS from Denmark (Pinholt et al. 2015) and Australia (Howden et al. 2013), which showed genetically similar strains in different geographical regions, suggesting this phenomenon is not restricted to the United Kingdom. Since transfer of patients between different hospitals may not fully explain the widespread dissemination, it may be the case that hospital-associated lineages are also carried in the community. Individuals can carry VRE for weeks to years after hospital discharge (Park et al. 2011; Karki et al. 2013; Sohn et al. 2013), and VRE can survive on environmental surfaces for at least 3 mo (Neely and Maley 2000), making transmission within the community a plausible possibility. Hospital-associated *E. faecium* has largely been considered to be restricted to the hospital setting. If factors outside of the hospital are involved, this would have important implications for infection prevention and control.

We showed that *E. faecium* isolates carrying *vanA* are genetically indistinguishable from VSE isolates at the core genome level, which is consistent with findings from Australia for *vanB* (Howden et al. 2013). This has important implications for infection control, since VSE isolates need to be considered in infection control initiatives targeting VRE, a suggestion also proposed by Howden et al. (2013) based on *vanB*-positive *E. faecium*. The findings in this study provide important additional insights over current knowledge by demonstrating that the dynamics of the *vanA*- and *vanB*-resistance determinants differ. This may relate to the predominant presence of *vanA* on plasmids compared with the integration and stable carriage of *vanB* in the chromosome together with a putative secondary fitness benefit provided by a bacteriocin associated with *vanB*. We also identified a *vanA*-positive isolate in Clade B, demonstrating that vancomycin resistance is not restricted to the hospital-associated lineage. While only a single case, this is important since the ability of vancomycin resistance to be acquired by other lineages has potentially serious implications for the future control of VRE.

Finally, we identified two *vanA* transposons with a higher prevalence in the East of England and in Ireland respectively, suggesting local circulation or a local source for *vanA*. Local circulation of *Tn1546* at the hospital level has been described (Kawalec et al. 2000, 2007; Naas et al. 2005; Lee et al. 2012), and geographical segregation of *Tn1546* has been reported by Schouten et al. (2001), who categorized *Tn1546* into two lineages dominating in different countries. Investigation into the dominant source(s) of *vanA* will be important, since it is repeatedly acquired by different lineages and may represent a target for infection control.

## Methods

### Isolates and antimicrobial susceptibility testing

The study was approved by the National Research Ethics Service (ref: 12/EE/0439) and the CUH Research and Development (R&D) department. Five hundred and fifteen isolates were obtained from NCTC (*n* = 11) and the BSAC (*n* = 504). The BSAC collection consisted of isolates submitted to a bacteremia resistance surveillance program (www.bsacsurv.org) between 2001 and 2011 from 40 hospital microbiology laboratories across UK&I. These were assigned to referral networks, defined as clusters of hospitals that are more likely to exchange patients within the cluster than with hospitals outside of that cluster (Donker et al. 2012). All VREfm isolates in the BSAC collection were obtained and sequenced (*n* = 256). A subset of 248 VSEfm were selected from the BSAC collection, matched where possible with a VRE isolate from the same hospital and year (*n* = 121), or hospital (*n* = 88). Nine VREfm isolates were subsequently excluded from further analysis for technical reasons, giving an overall total of 506 genomes taken forward into the analysis (for details, see Supplemental Table S2). The number of isolates by year of isolation for the BSAC collection is shown in Supplemental Figure S6. Antimicrobial-susceptibility testing was performed using the Vitek2 instrument with a P607 card (Biomerieux) for isolates provided by NCTC and using the agar dilution method for the BSAC collection (Andrews 2001).

### Whole-genome sequencing and data analysis

DNA extraction, sequencing, and assembly of reads were performed as previously described (Raven et al. 2016). Sequencing was performed on an Illumina HiSeq2000. Details of reads, depth of coverage, and N50 are provided in Supplemental Table S3. All isolates had greater than 74× coverage overall, and had a minimum of 73% of the genome with 50× coverage or higher. Genomes were assembled using Velvet (Zerbino and Birney 2008) with the improvements described previously (Page et al. 2016a). STs were identified from the sequence data using the MLST database (pubmlst.org/efaecium) and an in-house script (Supplemental File S1; https://github.com/sanger-pathogens/mlst_check). BAPS groups were defined based on the concatenated MLST genes, as described by Willems et al. (2012). STs identified in this study were cross-referenced with those described by Willems et al. (2012), and BAPS groups were assigned where there was overlap.

Study genomes were contextualized against a global collection. Sequence data for 73 *E. faecium* isolates reported previously (Lebreton et al. 2013) were downloaded from the ENA, combined with the 506 study genomes, and the 579 genomes were annotated using Prokka. The pangenome was estimated using Roary (Page et al. 2015), and an alignment was created of all core genes that were present in 99% of isolates. SNPs in the core genes were extracted (Page et al. 2016b) and used to construct a maximum likelihood tree using RAxML with 100 bootstraps and a midpoint root. Study genomes were related to the previous clade classification (Clades A1, A2, and B) by Lebreton et al. (2013). Genetic diversity was defined within and between the two clades based on pairwise SNP differences and excluded a single outlying isolate that resided between Clades A and B.

Four hundred and seventy-nine BSAC isolate genomes clustered within a single clonal expansion of Clade A. Sequence reads for 477/479 genomes were mapped to the *E. faecium* reference strain Aus0004 (ENA accession no. CP005531) using SMALT. Two isolates were excluded because identification of recombination events failed. Genetic regions were classed as mobile genetic elements if the annotation predicted phage-, plasmid-, IS-, or transposon-related genes or if PHAST (Zhou et al. 2011) identified a putative prophage (phast.wishartlab.com). These regions plus recombination identified using Gubbins (Croucher et al. 2014) were removed from the genomes mapped to the reference genome to create a “core” genome (Supplemental File S2; https://github.com/sanger-pathogens/remove_blocks_from_aln). SNPs in this core genome were used to create maximum likelihood phylogenies using RAxML with 100 bootstraps and a midpoint root. Trees were visualized using FigTree and iTOL (Letunic and Bork 2007, 2011).

A phylogenetic comparison was made between MLST and WGS. MLST loci were concatenated and aligned, and a RAxML tree was constructed with 100 bootstraps and a midpoint root. This was compared to the maximum likelihood tree based on SNPs in the core genome using Dendroscope (Huson et al. 2007; Huson and Scornavacca 2012). A conservative estimate of the number of discrepancies between WGS and MLST trees was made based on the following assumptions. The largest cluster for each ST was assumed to be the correct assignment, as were STs containing a single isolate. The exceptions were ST17, ST18, and ST203, which were each distributed throughout a single branch of the tree but intermixed with other STs. This distribution suggested that these could be the basal ST for the branch, and therefore, all of the isolates on the branch with that ST were assumed to be in the correct position. A discrepancy was defined when one or more isolates resided on a branch of the tree that was genetically distinct from the presumed correct position. The genetic basis for discrepancies were ascribed to recombination or mutation through a manual analysis of the sequence data. A mutation in an MLST locus was defined when there was a single SNP in the locus compared with the sequence of the locus in surrounding isolates. A recombination event over an MLST locus was suspected when there was more than one SNP in the locus compared with the sequence of the locus in surrounding isolates. Suspected recombination events were then verified through analysis of recombination regions across the entire genome, identified using Gubbins (Croucher et al. 2014). Recombination events over the MLST loci were mapped against the phylogeny to determine whether a recombination event had occurred over one or more of the relevant MLST loci in the branch leading to the discrepant ST(s). Homologs of the MLST locus *pstS* were identified by comparing the *E. faecalis pstS*1 (the basis for the *E. faecium* MLST scheme) and the *E. faecium* Aus0004 genome using BLASTN.

### Vancomycin resistance

The presence of *vanA* and *vanB* were established by in silico PCR using published primers (Dutka-Malen et al. 1995; Depardieu et al. 2004). Sequence reads were mapped to a relevant reference using SMALT (*Tn1546* [ENA accession no. M97297] from *E. faecium* strain BM4147 for *vanA*, and Aus0004 reference genome [2835430–2869240 bp] for *vanB*). Regions of sequence upstream of and downstream from the *vanA* and *vanB* transposons were identified as previously described (Howden et al. 2013). Insertion sites were identified as follows. *vanB*, regions of up to 10,000 bp were identified upstream of and downstream from the *vanB* transposon and compared using BLASTN; *vanA*, the last 734 bp (*vanZ* plus inverted repeat) of *Tn1546* and the adjacent sequence up to 5000 bp for a single representative of each unique 5000 bp was compared with a local database of all 506 assemblies using WebACT. Variations in the *vanA* and *vanB* transposons were determined by identifying SNPs compared to the reference and by comparing the regions of the reference transposon that were present/absent.

### Virulence genes

Putative *E. faecium* virulence genes in the 506 study genomes were identified from the VFDB (http://www.mgc.ac.cn/VFs/). Gene presence was detected by in silico PCR using previously published primers, as follows: *acm* (Nallapareddy et al. 2003), *ebpA* (Sillanpää et al. 2010), *ecbA* (Soheili et al. 2014), *esp* (Vankerckhoven et al. 2004), *scm* (Soheili et al. 2014), *sgrA* (Soheili et al. 2014), *hyl* (Vankerckhoven et al. 2004), and *efa* (Eaton and Gasson 2001).

## Data access

Sequence data from this study have been submitted to the European Nucleotide Archive (ENA; http://www.ebi.ac.uk/ena) under the accession numbers listed in Supplemental Table S2.

## Supplementary Material

Supplemental Material
