# Supplementary material for: A decade of genomic history for healthcare-associated Enterococcus faecium in the United Kingdom and Ireland
Source: Genome Res. 2016 Oct;26(10):1388–96. doi: 10.1101/gr.204024.116 (PMC5052055; doi:10.1101/gr.204024.116)
Supplement: Supplemental Material [file supp_26_10_1388__index.html]

A decade of genomic history for healthcare-associated Enterococcus faecium in the United Kingdom and Ireland — Supplemental Material 

# A decade of genomic history for healthcare-associated *Enterococcus faecium* in the United Kingdom and Ireland

## Supplemental Material

**Files in this Data Supplement:**

- Supplemental\_Table\_S3.pdf
- Supplemental Data.pdf
- Supplemental Table S2.xlsx
- Supplemental\_File\_S1.zip
- Supplemental\_File\_S2.zip
