# Supplementary material for: A decade of genomic history for healthcare-associated Enterococcus faecium in the United Kingdom and Ireland
Source: Genome Res. 2016 Oct;26(10):1388–96. doi: 10.1101/gr.204024.116 (PMC5052055; doi:10.1101/gr.204024.116)
Supplement: Supplemental Material [file supp_gr.204024.116_Supplemental_Table_S3.pdf]

Isolate ID	Reads	Depth of Coverage	N50
BSAC_ec1016	3929856	122.26	52733
BSAC_ec1031	3834498	100.36	77598
BSAC_ec1032	3957952	109.9	48065
BSAC_ec1033	3516928	95.06	48296
BSAC_ec1034	4196206	110.45	82860
BSAC_ec1057	4146314	129.66	53172
BSAC_ec1061	3525570	101.61	45514
BSAC_ec1063	3771146	99.31	45610
BSAC_ec1065	5172006	145.62	44308
BSAC_ec1066	3949484	116.52	53082
BSAC_ec1067	3728828	108.35	50944
BSAC_ec1071	3765564	104.59	49300
BSAC_ec1072	3462380	90.97	51982
BSAC_ec1073	3840664	98.91	42370
BSAC_ec1074	4303996	102.81	59079
BSAC_ec1075	4019974	112.52	52803
BSAC_ec1076	3682404	94.04	58205
BSAC_ec1077	3836616	109.45	41611
BSAC_ec1081	3762010	107.8	47885
BSAC_ec1085	3991900	112.48	47892
BSAC_ec1090	3466432	82.92	47698
BSAC_ec1098	4268206	111.13	54896
BSAC_ec1103	3933310	117.5	60582
BSAC_ec1106	4050236	127.08	46338
BSAC_ec1111	3811762	104.06	73628
BSAC_ec1112	5024960	146.66	69745
BSAC_ec1116	3902776	107.36	65517
BSAC_ec1119	4180304	128.03	48237
BSAC_ec1123	4068118	110.62	60199
BSAC_ec1137	3765300	107.56	55712
BSAC_ec1140	3925390	113.87	52728
BSAC_ec1142	3745836	100.05	43698
BSAC_ec1144	3876508	106.02	42690
BSAC_ec1146	3745182	108.58	51681
BSAC_ec1148	4412416	113.6	40842
BSAC_ec1150	4488520	127.82	50292
BSAC_ec1153	3930586	97.21	64383
BSAC_ec1154	3819616	112.61	38814
BSAC_ec1161	3459800	81.14	56434
BSAC_ec1162	3959070	101.78	58198
BSAC_ec1163	4617888	120.29	60261
BSAC_ec1167	4389826	105.98	52715
BSAC_ec1168	3471230	99.43	56579
BSAC_ec1177	3982840	106.52	61443
BSAC_ec1179	4012276	112.91	52736

BSAC_ec1180	4150138	110.33	53308
BSAC_ec1201	4201950	108.63	39284
BSAC_ec1203	4122432	125.01	47908
BSAC_ec1205	3869458	107.48	43410
BSAC_ec1207	4224956	102.64	39257
BSAC_ec1212	4695440	138.23	39330
BSAC_ec1217	3580788	103.84	48869
BSAC_ec1223	3825410	104.91	51838
BSAC_ec1227	4223538	117.75	55332
BSAC_ec1231	4358022	109.28	40809
BSAC_ec1232	3569078	104.23	164118
BSAC_ec1234	3673364	108.55	53538
BSAC_ec1241	3512686	94.56	46443
BSAC_ec1242	3684276	94.41	66230
BSAC_ec1244	3880480	107.52	45374
BSAC_ec1245	3762098	102.1	48948
BSAC_ec1248	4882576	144.06	46346
BSAC_ec1250	4572400	126.27	51453
BSAC_ec1267	3661424	101.64	60446
BSAC_ec1272	3898806	112.19	43403
BSAC_ec1281	3522400	96.05	57937
BSAC_ec1286	3704462	113.84	49316
BSAC_ec1287	3500406	102.17	59575
BSAC_ec1288	3788766	101.21	66236
BSAC_ec1301	3883780	113.78	56576
BSAC_ec1307	3442974	89.24	50138
BSAC_ec1310	4294036	108.53	46292
BSAC_ec1313	3936098	106.8	36568
BSAC_ec1322	4774786	128.65	64817
BSAC_ec1324	4961266	125.56	55432
BSAC_ec1325	3542580	98.71	46308
BSAC_ec1326	3815780	101.59	44064
BSAC_ec1327	4157420	101.65	55838
BSAC_ec1331	5619344	162.17	50564
BSAC_ec1338	3884988	115.34	43237
BSAC_ec1339	4095288	117.53	60119
BSAC_ec1340	3725026	109.79	40818
BSAC_ec1349	4049094	126.25	48248
BSAC_ec1352	3592048	103.18	54648
BSAC_ec1353	3878782	102.61	53744
BSAC_ec1356	4374248	118.87	57945
BSAC_ec1357	4058556	108.79	59809
BSAC_ec1358	4029448	106.33	59609
BSAC_ec1359	3698480	97.59	59793
BSAC_ec1361	4210856	113.74	47192
BSAC_ec1369	3499694	97.52	40577

BSAC_ec1382	5101894	146.22	50568
BSAC_ec1388	3905380	116.76	47523
BSAC_ec1392	3813740	108.72	50295
BSAC_ec1394	3660242	99.26	35766
BSAC_ec1395	3412916	97.25	48956
BSAC_ec1399	3794972	98.7	50270
BSAC_ec1406	4083558	108.81	49303
BSAC_ec1416	3922928	119.08	52803
BSAC_ec142	3869366	109.73	46342
BSAC_ec1426	3641662	101.29	51840
BSAC_ec143	3582532	98.15	50494
BSAC_ec1430	3606786	95.27	52805
BSAC_ec1439	4023900	110.06	55140
BSAC_ec144	4015408	114.22	49550
BSAC_ec1451	3409320	92.35	53351
BSAC_ec1452	3636770	101.01	42590
BSAC_ec1454	3671072	91.26	41234
BSAC_ec1455	3898688	110.55	48386
BSAC_ec1483	3796280	112.36	49268
BSAC_ec1484	3658694	102.24	47312
BSAC_ec1485	4445178	124.25	47545
BSAC_ec1489	4088204	117.6	48845
BSAC_ec1491	3948412	110.37	49279
BSAC_ec1496	3394188	93.93	49340
BSAC_ec1497	3422856	104.15	54020
BSAC_ec1498	3897482	113.45	32526
BSAC_ec15	4049750	113.15	77973
BSAC_ec1512	3958468	104.32	44894
BSAC_ec1514	3940596	114.61	38990
BSAC_ec1563	4300624	132.05	49303
BSAC_ec1564	3496822	93.19	249351
BSAC_ec1565	3659146	95.2	48918
BSAC_ec1568	3804824	87.2	54870
BSAC_ec1570	4202348	124.02	48960
BSAC_ec1572	4256120	118.64	43436
BSAC_ec1576	4315532	98.86	49723
BSAC_ec1577	3702906	96.36	44061
BSAC_ec1582	3372420	99.45	46999
BSAC_ec1589	4264438	114.96	53757
BSAC_ec1602	4197180	119.99	50905
BSAC_ec1603	3824524	109.47	49835
BSAC_ec1608	4498700	136.17	45405
BSAC_ec1609	5279160	139.5	53905
BSAC_ec1618	4028948	112.68	170657
BSAC_ec162	4486478	115.22	49349
BSAC_ec1621	4146396	103.97	46901

BSAC_ec1626	3657732	109.53	51334
BSAC_ec1628	3738854	109.9	48146
BSAC_ec1629	4096722	111.96	38346
BSAC_ec163	3741596	107.24	58272
BSAC_ec1651	3772348	90.92	47883
BSAC_ec1653	4084164	114.09	48299
BSAC_ec1658	3984064	115.45	49454
BSAC_ec1660	5606218	164.23	48846
BSAC_ec1662	3417108	90.85	56036
BSAC_ec1666	3863274	108.28	56938
BSAC_ec1671	3918200	111.32	62324
BSAC_ec1684	3887656	103.43	82229
BSAC_ec1687	3865444	100.84	52447
BSAC_ec1688	3727564	104.07	44761
BSAC_ec1693	3788530	105.73	51562
BSAC_ec1694	3479082	97.65	47309
BSAC_ec1699	5303718	156.35	62138
BSAC_ec1701	4117552	114.08	53371
BSAC_ec1702	3807504	114.8	52466
BSAC_ec1703	4996758	130.3	54602
BSAC_ec1706	3649578	95.52	52322
BSAC_ec1707	4642676	124.29	52344
BSAC_ec1708	3775284	99.99	53887
BSAC_ec1715	5267588	134.58	48710
BSAC_ec1717	5768284	159.37	38694
BSAC_ec1723	4426956	116.6	48957
BSAC_ec1725	5435010	153.44	40871
BSAC_ec1729	3673830	99.84	51425
BSAC_ec1732	4134758	122.02	68874
BSAC_ec1746	4084950	116.52	48947
BSAC_ec1801	3653340	106.08	50400
BSAC_ec1802	3717008	108.7	74866
BSAC_ec1806	3837924	103.75	50334
BSAC_ec1809	4063748	109.31	37848
BSAC_ec1814	3834644	100.33	59346
BSAC_ec1815	3882566	105.16	50266
BSAC_ec1821	3570846	90.96	54112
BSAC_ec1822	3675506	90.91	50611
BSAC_ec1824	3807636	106.44	36218
BSAC_ec1825	3903902	117	49319
BSAC_ec1829	3873222	100.89	49731
BSAC_ec1840	3799308	104.08	54073
BSAC_ec1902	3560598	92.49	49300
BSAC_ec1903	3975894	100.55	49350
BSAC_ec1906	3608398	96.95	46730
BSAC_ec1908	3619704	97.58	49322

BSAC_ec1910	3567856	94.49	40944
BSAC_ec1935	3412236	100.5	43523
BSAC_ec1937	4068076	117.12	66019
BSAC_ec1949	4873178	134.59	48904
BSAC_ec1951	3828710	105.08	57742
BSAC_ec1952	3708806	98.88	54154
BSAC_ec1971	3783750	107.43	41256
BSAC_ec1972	4022366	99.83	50256
BSAC_ec1973	3960414	103.87	39288
BSAC_ec1984	3637926	102.51	43083
BSAC_ec1993	4039158	112.03	59579
BSAC_ec1997	4076550	100.8	53889
BSAC_ec1998	3777366	111.25	68909
BSAC_ec2000	3888070	92.91	55828
BSAC_ec2004	4021742	115.01	54380
BSAC_ec2005	3502598	96.74	53003
BSAC_ec2006	4000636	105.12	35012
BSAC_ec2008	4031322	105.72	49752
BSAC_ec2009	3617622	96.93	56566
BSAC_ec2032	3537170	101.38	93647
BSAC_ec2034	3881668	102.18	52107
BSAC_ec2036	3998384	120.44	48379
BSAC_ec2037	4096098	122.88	50993
BSAC_ec2041	3443636	95.42	58785
BSAC_ec2043	3728882	109.01	53427
BSAC_ec2044	4864650	132.95	47928
BSAC_ec2048	3970040	100.25	50923
BSAC_ec2050	3264154	96.2	44015
BSAC_ec2054	3651406	105.35	64171
BSAC_ec2061	5003324	132.9	49762
BSAC_ec2062	3449008	93.84	36646
BSAC_ec2065	3753708	97.78	50193
BSAC_ec2070	3367126	87.94	53891
BSAC_ec2071	3550334	94.8	61995
BSAC_ec2074	3537976	96.08	49257
BSAC_ec2075	3854654	118.39	73657
BSAC_ec2076	4331366	123.41	45612
BSAC_ec2077	3586374	101.04	74996
BSAC_ec2078	4457792	106.17	53890
BSAC_ec2080	3947096	96.41	59859
BSAC_ec2084	3848206	102.81	59052
BSAC_ec2085	4118432	122.99	47091
BSAC_ec2087	3993476	112.99	39012
BSAC_ec2090	4138036	104.34	53929
BSAC_ec2101	4248254	118.56	44785
BSAC_ec2114	3829186	111.92	61329

BSAC_ec2124	4165772	115.5	37074
BSAC_ec2125	4386742	130.32	50137
BSAC_ec2127	4761520	138.8	47932
BSAC_ec2129	4334928	124.07	47887
BSAC_ec2133	4296722	114.23	65022
BSAC_ec2149	4413758	128.59	66356
BSAC_ec2150	3835944	104.21	64288
BSAC_ec2155	4499718	128.56	53214
BSAC_ec2183	3850908	103.86	55983
BSAC_ec2184	4519516	131.21	78082
BSAC_ec2194	3806016	105.41	47306
BSAC_ec2198	3869220	92.44	53029
BSAC_ec2201	4095560	111.64	57948
BSAC_ec2204	3999456	103.69	39388
BSAC_ec2210	3919580	112.95	40981
BSAC_ec222	3723764	114.93	278715
BSAC_ec2220	3310796	88.44	49750
BSAC_ec223	3540916	94.79	41902
BSAC_ec224	4064614	109.53	52873
BSAC_ec225	3792404	100.14	51838
BSAC_ec2259	3367172	101.3	37848
BSAC_ec2268	3646960	105.77	50461
BSAC_ec227	4176110	126.6	53284
BSAC_ec2271	3853934	99.02	61875
BSAC_ec2281	3463312	91.3	58790
BSAC_ec2284	3797808	115.36	45388
BSAC_ec2285	4647348	113.66	53907
BSAC_ec2286	3826998	99.86	50069
BSAC_ec2288	3755588	100.36	50371
BSAC_ec229	3771418	100.39	49551
BSAC_ec2290	3956490	105.48	51729
BSAC_ec2293	3865252	106.75	47159
BSAC_ec2294	4145694	129.49	53782
BSAC_ec2298	3904604	112.14	66968
BSAC_ec2299	3852080	109.94	61149
BSAC_ec2300	3977562	103.73	50085
BSAC_ec2306	3684264	97.01	50037
BSAC_ec2308	3926490	91.78	59870
BSAC_ec2310	4143814	117.75	48149
BSAC_ec2311	3846216	114.93	48910
BSAC_ec2312	3979060	117.59	49758
BSAC_ec2314	3557106	89.24	53905
BSAC_ec2318	3753470	105.3	58520
BSAC_ec2329	4022680	105.4	60832
BSAC_ec2334	3209702	88.81	58274
BSAC_ec2335	3294700	86.64	44052

BSAC_ec2338	4083458	122.64	38138
BSAC_ec2340	3898244	107.51	60911
BSAC_ec2341	3994834	119.41	50968
BSAC_ec2346	3739166	102.36	51440
BSAC_ec2351	4357018	127.54	48972
BSAC_ec2352	4444434	114.71	53763
BSAC_ec2353	3688792	103.08	52679
BSAC_ec2355	3733112	94.56	48925
BSAC_ec2356	3487752	101.58	51792
BSAC_ec2357	2901134	74.12	56301
BSAC_ec2364	4129774	109.12	56579
BSAC_ec2365	3803046	102.61	49283
BSAC_ec2380	3655890	96.31	55805
BSAC_ec2382	3758082	96.36	40634
BSAC_ec239	4391754	132.37	52803
BSAC_ec2396	4206020	119.12	44430
BSAC_ec2409	3824916	108.66	45590
BSAC_ec241	3446182	99.73	49319
BSAC_ec2411	3884458	106.21	56250
BSAC_ec242	3880302	114.04	74185
BSAC_ec2420	4018396	111.01	52805
BSAC_ec2441	4086348	116.2	53915
BSAC_ec2442	3413958	99.19	59385
BSAC_ec2443	3681124	102.89	59361
BSAC_ec2444	3966580	118.96	47307
BSAC_ec2446	5010722	146.57	64713
BSAC_ec245	3473942	95.92	44051
BSAC_ec246	3174208	95.06	51435
BSAC_ec2482	4681528	126.6	43397
BSAC_ec2484	3685522	106.39	49352
BSAC_ec2485	3839534	104.99	65872
BSAC_ec2486	4537300	117.69	49500
BSAC_ec2496	4258382	109.57	56319
BSAC_ec2498	3369688	104.42	101645
BSAC_ec2499	4129348	116.42	41265
BSAC_ec2500	3961466	111.36	52812
BSAC_ec2502	3916732	99.51	59574
BSAC_ec2503	5310464	155.31	53919
BSAC_ec251	4091792	109.47	52665
BSAC_ec2511	4018490	127.06	49307
BSAC_ec2513	4064400	109.94	53690
BSAC_ec2515	3345808	83.68	37996
BSAC_ec2516	3780650	104.48	66082
BSAC_ec2529	3634390	99.41	48178
BSAC_ec2532	4260146	127.97	68874
BSAC_ec2535	4373044	121.05	63234

BSAC_ec2559	3582042	105.79	81166
BSAC_ec256	3203950	95.99	55428
BSAC_ec2561	3876872	111.04	53007
BSAC_ec2562	3775366	101.12	41858
BSAC_ec2565	3942844	113.7	57437
BSAC_ec2566	3994594	112.27	52326
BSAC_ec2569	4236256	115.3	62829
BSAC_ec2570	3788056	90.75	45699
BSAC_ec2572	3597884	105.41	50476
BSAC_ec2573	3906692	105.87	59136
BSAC_ec2575	4905948	137.15	52684
BSAC_ec2577	3640716	102.92	56429
BSAC_ec2578	3586462	91.08	55993
BSAC_ec2579	4177614	107.95	55703
BSAC_ec2594	4532610	136.23	57847
BSAC_ec2596	4447120	127.01	43332
BSAC_ec2597	4223618	100.44	39104
BSAC_ec2599	4097426	117.33	44028
BSAC_ec2600	3900394	88.98	49760
BSAC_ec2610	4396350	112.58	56213
BSAC_ec2611	3215024	89.76	49857
BSAC_ec2614	4875306	132.2	48539
BSAC_ec2622	3869920	112.66	56588
BSAC_ec2629	3797320	92.94	43242
BSAC_ec263	3955044	105.68	48873
BSAC_ec2631	4592592	127.83	50294
BSAC_ec2632	3200068	91.08	50296
BSAC_ec2633	3784716	107.61	68900
BSAC_ec2641	4049244	112.63	55112
BSAC_ec2644	4478786	118.79	54140
BSAC_ec2647	3126150	78.48	66255
BSAC_ec265	3646394	98.9	50865
BSAC_ec2660	4037820	109.73	53188
BSAC_ec2668	3207204	93.16	66255
BSAC_ec2685	3791452	92.86	34319
BSAC_ec2690	3870170	97.74	34702
BSAC_ec2700	3862808	110.98	59565
BSAC_ec2701	3856702	108.98	61156
BSAC_ec2708	3955452	114.22	48094
BSAC_ec2727	4687788	132.7	49766
BSAC_ec2729	4352844	125.83	55256
BSAC_ec2731	3944678	107.4	50159
BSAC_ec2732	3574768	88.54	41162
BSAC_ec2733	3470816	90.03	49494
BSAC_ec2742	3751602	116.2	49973
BSAC_ec2744	3412444	96.65	48903

BSAC_ec2745	3785218	92.4	47908
BSAC_ec2750	3830974	113.11	55927
BSAC_ec2755	4548490	136.51	56574
BSAC_ec2759	3426748	91.24	40599
BSAC_ec2761	4140662	121.56	61235
BSAC_ec2783	3668200	104.69	121955
BSAC_ec2791	3778832	100.67	49080
BSAC_ec289	4707996	125.62	49687
BSAC_ec293	3888090	115.26	61804
BSAC_ec295	3786606	99.6	56408
BSAC_ec296	4266916	133.72	51452
BSAC_ec300	4744666	118.2	49347
BSAC_ec311	3392878	94.6	89363
BSAC_ec312	4128506	122.65	44166
BSAC_ec314	4331034	127.49	45378
BSAC_ec316	4027364	94.89	59575
BSAC_ec318	4722926	137.2	58221
BSAC_ec32	3996206	123.65	68693
BSAC_ec332	3961666	108.36	63489
BSAC_ec335	3648590	104.99	48856
BSAC_ec336	3981104	99.91	45380
BSAC_ec337	4189666	108.32	70518
BSAC_ec34	4156728	122.7	56561
BSAC_ec340	3617448	99.07	46319
BSAC_ec342	4005396	125.12	51434
BSAC_ec356	4138766	120.82	52802
BSAC_ec361	4069474	122.8	53169
BSAC_ec368	3880006	112.94	52368
BSAC_ec383a	4781308	143.31	51436
BSAC_ec384	4038462	109.08	47893
BSAC_ec391	4711534	129.18	46751
BSAC_ec411	3500228	106.93	65642
BSAC_ec412	3932716	107.02	287976
BSAC_ec421	3839302	117.94	53379
BSAC_ec426	3813056	108.88	53167
BSAC_ec472	3556050	96.93	58774
BSAC_ec474	3896996	108.69	51855
BSAC_ec476	3621682	92.01	48945
BSAC_ec479	4047986	114.4	49580
BSAC_ec493	3426386	105.42	52541
BSAC_ec494	3645408	106.05	47911
BSAC_ec495	3914786	110.69	52382
BSAC_ec514	3864790	97.77	51821
BSAC_ec516	3996832	112.56	60993
BSAC_ec519	4142510	112.95	53817
BSAC_ec531	3590266	100.27	36849

BSAC_ec533	3757214	109.35	49271
BSAC_ec537	3875688	110.84	48904
BSAC_ec538	3800630	98.79	59719
BSAC_ec547	3905424	123.63	122324
BSAC_ec548	4398878	133.69	49357
BSAC_ec555	3811582	100.4	299148
BSAC_ec562	4532154	118.8	57932
BSAC_ec565	3647216	91.65	34703
BSAC_ec566	3955038	112.19	49540
BSAC_ec570	3862678	107.89	48564
BSAC_ec571	3654016	93.97	94920
BSAC_ec577	3335426	101.5	53421
BSAC_ec578	3614882	110.2	52803
BSAC_ec582	4207230	125.91	58221
BSAC_ec587	3936612	104.07	56260
BSAC_ec603	3654768	106.75	45366
BSAC_ec604	3918350	114.57	44758
BSAC_ec650	3535678	98.86	57987
BSAC_ec66	3944938	102.05	58319
BSAC_ec667	4326124	132.29	59575
BSAC_ec668	3941840	117.33	48664
BSAC_ec67	3756022	110.66	48617
BSAC_ec673	3194960	97.47	54153
BSAC_ec675	3862244	117.93	52733
BSAC_ec676	4758514	125.68	58217
BSAC_ec68	4019410	115.23	53323
BSAC_ec680	3610974	95.67	44943
BSAC_ec698	3889624	106.76	52886
BSAC_ec71	3646656	100.2	52803
BSAC_ec72	3514298	106.32	56773
BSAC_ec725	3728812	93.91	53115
BSAC_ec728	3977916	115.31	45975
BSAC_ec73	3713606	103.67	56575
BSAC_ec730	3852794	118.19	53315
BSAC_ec739	4048946	124.83	265265
BSAC_ec747	3423014	105.21	53520
BSAC_ec748	3661786	105.97	58853
BSAC_ec752	3807536	104.76	55215
BSAC_ec754	3939542	126.61	49321
BSAC_ec781	3861716	115.37	65012
BSAC_ec782	3806362	109.76	64394
BSAC_ec786	4164920	121.1	60580
BSAC_ec787	3660736	105.56	34944
BSAC_ec79	3281872	90.05	63672
BSAC_ec790	3566048	100.04	66236
BSAC_ec80	3737638	108.93	48906

BSAC_ec809	4256216	108.66	48090
BSAC_ec814	3920938	96.19	41187
BSAC_ec816	3750422	109.36	37298
BSAC_ec819	4150830	117.95	47891
BSAC_ec822	3779426	86.49	42696
BSAC_ec823	4081156	115.75	52803
BSAC_ec824	3671252	99.14	52803
BSAC_ec827	3683776	96.89	47873
BSAC_ec828	3668858	111.34	50983
BSAC_ec832	3697158	102.78	48920
BSAC_ec833	3996624	107.33	41106
BSAC_ec835	4084944	116	69185
BSAC_ec837	4419752	119.41	34755
BSAC_ec852	5112848	153.81	42960
BSAC_ec860	3540928	110.98	49290
BSAC_ec871	3971370	119.81	67655
BSAC_ec893	3872940	121.45	97471
BSAC_ec902	3324870	92.3	47891
BSAC_ec903	4133200	120.29	50498
BSAC_ec908	3754988	112.19	45378
BSAC_ec909	3557242	106.65	53520
BSAC_ec911	4380312	115.61	59737
BSAC_ec917	4259744	118.83	52810
BSAC_ec926	4626722	130.49	87950
BSAC_ec929	3479380	95.27	65408
BSAC_ec951	3729206	104.93	52663
BSAC_ec955	4050360	113.08	131945
BSAC_ec956	4169232	106.91	40809
BSAC_ec958	3517722	92.32	43006
BSAC_ec959	4235264	119.45	49305
BSAC_ec960	3619152	100.06	51325
BSAC_ec967	3547044	110.18	69629
BSAC_ec982	4068378	120.79	52460
BSAC_ec986	3626292	92.28	49328
BSAC_ec987	3846864	108.4	41423
BSAC_ec994	4907376	139.98	38923
NCTC 12202	3770318	107.67	70083
NCTC 12204	3708514	100.16	66031
NCTC 12952	3902366	109.78	70522
NCTC 13169	3812876	110.73	101722
NCTC 7171	3813730	122.6	206626
NCTC 7174	3767750	119.85	108107
NCTC 7176	3421362	110.35	191590
NCTC 7378	3501594	99.01	139735
NCTC 7379	3916604	115.85	270692
NCTC 7380	3505966	104.76	167195

NCTC 8619	3200348	100.16	84461
