# Supplementary material for: A decade of genomic history for healthcare-associated Enterococcus faecium in the United Kingdom and Ireland
Source: Genome Res. 2016 Oct;26(10):1388–96. doi: 10.1101/gr.204024.116 (PMC5052055; doi:10.1101/gr.204024.116)
Supplement: Supplemental Material [file supp_gr.204024.116_Supplemental_Data.pdf]

**A decade of genomic history for healthcare-associated *Enterococcus faecium* in the United Kingdom and Ireland**

Kathy E. Raven, Sandra Reuter, Rosy Reynolds, Hayley J. Brodrick, Julie E Russell, M. Estée Török, Julian Parkhill, Sharon J. Peacock.

**Supplementary Data**

Table of contents:

Supplemental Figure S1……………………………………………………………….…..2
Supplemental Figure S2……………………………………………………………….…..3
Supplemental Figure S3……………………………………………………………….…..4
Supplemental Figure S4……………………………………………………………….…..5
Supplemental Figure S5……………………………………………………………….…..6
Supplemental Figure S6……………………………………………………………….…..7
Supplemental Figure S7………………………………………………………………….....8
Supplemental Table S1…………………………………………………………………9

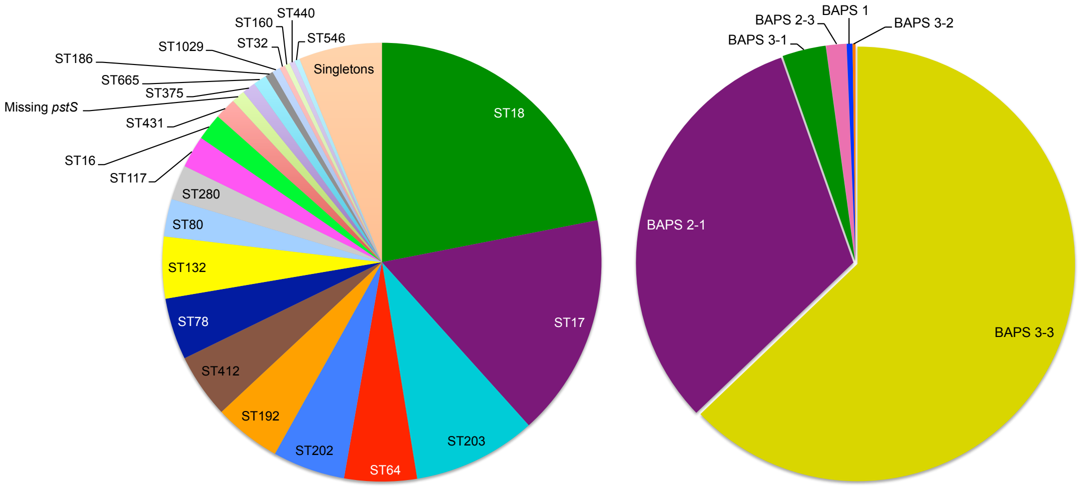


**Supplemental Figure S1**
Prevalence of STs and BAPs groups in the collection.

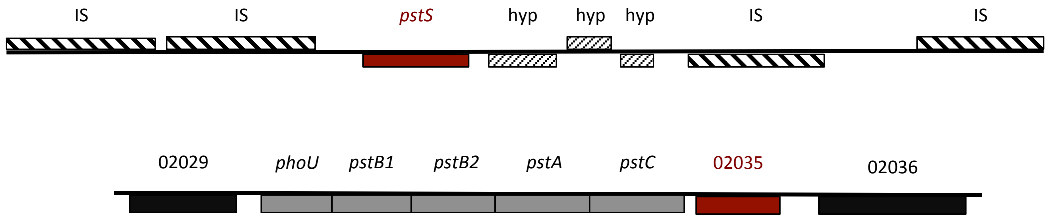


**Supplemental Figure S2**
Location of the *pstS* gene used in the MLST scheme (top diagram) and its homolog in the Aus0004 reference genome (02035) (bottom diagram). Genes highlighted in grey in the bottom diagram are described as being in the same operon as a phosphate binding protein (*pstS*) and together forming a complex involved in phosphate import. hyp = hypothetical protein.

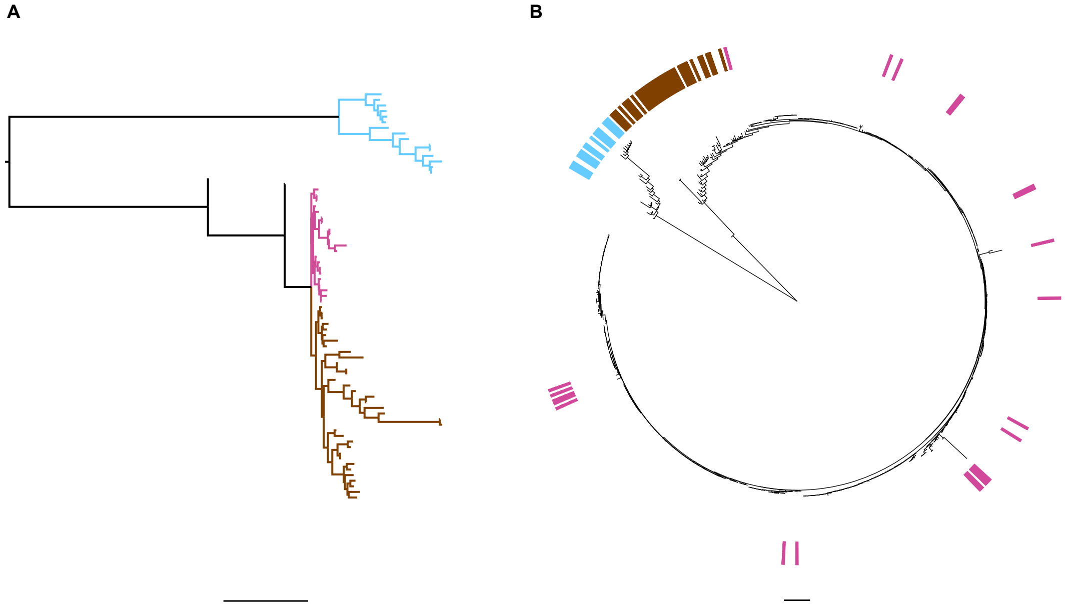


**Supplemental Figure S3**

A) Maximum likelihood tree based on SNPs in the core genes of 73 isolates reported by Lebreton *et al*. (2013). Colored branches indicate isolates belonging to Clades A1 (pink), A2 (brown) and B (blue), as defined by Lebreton *et al*. (2013). Scale bar, ~15,400 SNPs. B) Maximum likelihood tree based on SNPs in the core genes of the 506 isolates from this study and 73 isolates reported by Lebreton *et al*. (2013), where the definition for core genes was presence in 100% of isolates. Colored ring indicates isolates from Clades A1 (pink), A2 (brown) and B (blue) as defined by Lebreton *et al.* (2013). Scale bar, ~13,400 SNPs.

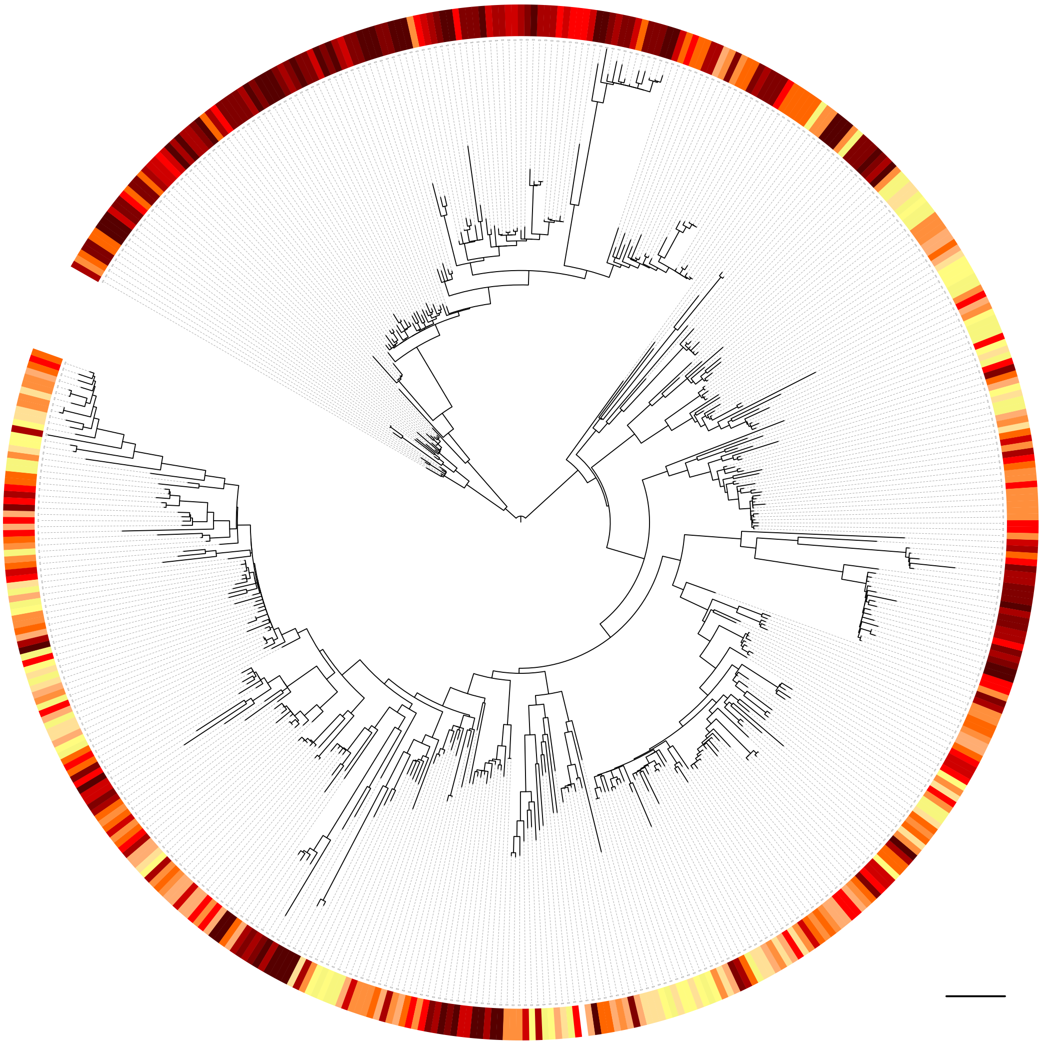

**Supplemental Figure S4**
Temporal distribution of *E. faecium* lineages across the UK and Ireland. Maximum likelihood tree based on SNPs in the core genome for national isolates belonging to the clonal expansion of Clade A. Colors shown in the circle represent year of isolation grading from 2001 (yellow) to 2011 (dark red). Scale bar, 90 SNPs.

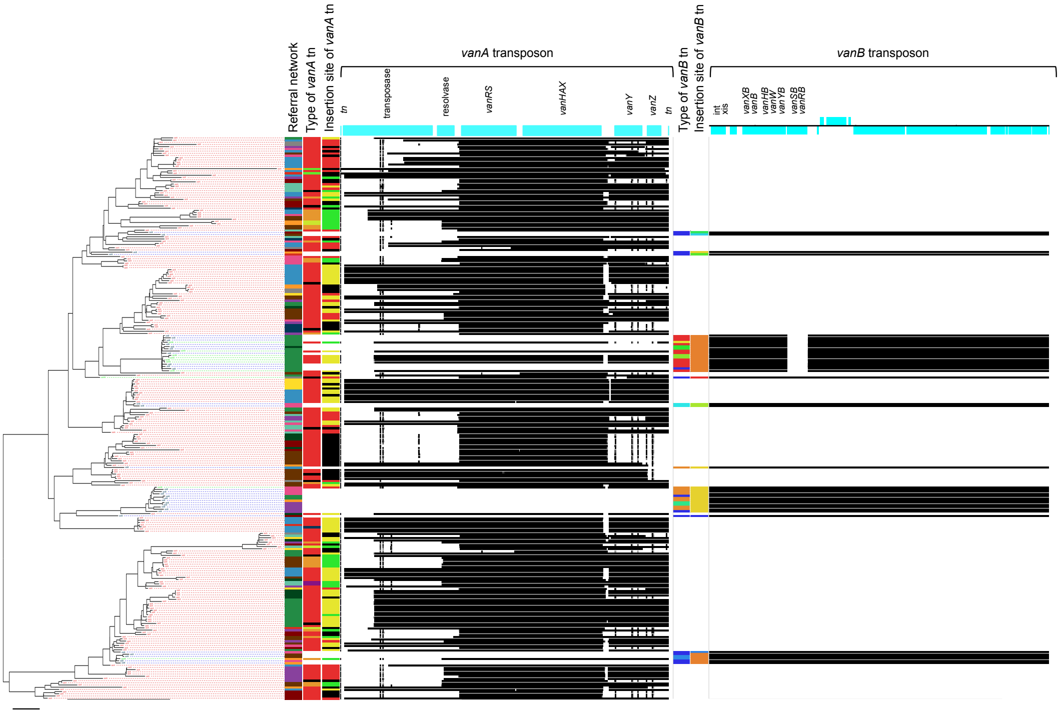


**Supplemental Figure S5**

Variants of *vanA* and *vanB* transposons. Left hand side: Maximum likelihood tree of the 257 v*anA* or *vanB* positive BSAC isolates from the clonal expansion of Clade A. Right hand side: Vertical bars indicate referral networks, sequence of the *vanA* and *vanB* transposons (unique sequences = black), and the insertion site of the *vanA* (best matches to pIP816=red, pLG1=yellow, pLG1/pF856/p5753cA/pS177=green; insufficient sequence=black) and *vanB* transposons. Horizontal bars indicate genetic content (present=black, absent=white) of the *vanA* and *vanB* transposons with a map of genes (top, turquoise blocks). Scale bar, 66 SNPs.

A

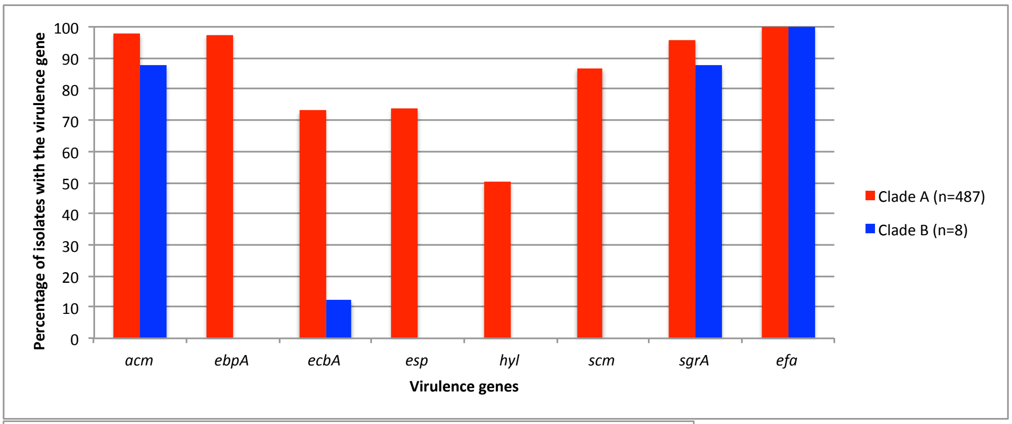


B

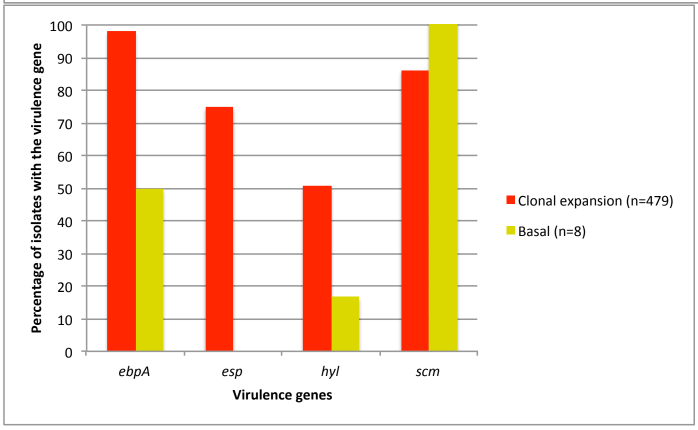


**Supplemental Figure S6**

A) Prevalence of virulence factors in the clinical isolates of Clade A and Clade B.

B) Prevalence of virulence factors in the basal and clonal expansion populations of Clade A, which are absent in Clade B.

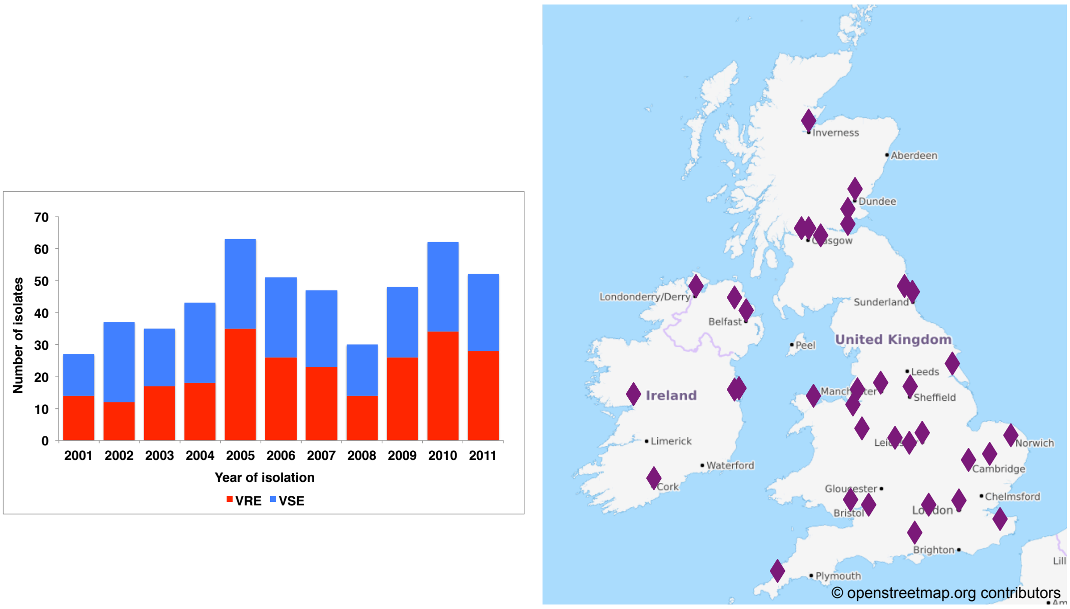


**Supplemental Figure S7**

Geographical and temporal distribution of the BSAC isolates.

| Pangenome annotation | Number of vanB positive isolates (n=34) |
|---|---|
| D-alanine—D-lactate ligase | 34 |
| Vancomycin B-type resistance protein VanW | 34 |
| D-specific alpha-keto acid dehydrogenase | 34 |
| D-alanyl-D-alanine carboxypeptidase | 34 |
| D-alanyl-D-alanine dipeptidase | 34 |
| integrase | 34 |
| Excisionase | 34 |
| DNA-directed RNA polymerase specialized sigma subunit | 34 |
| Site-specific DNA-methyltransferase (adenine-specific) | 34 |
| Relaxase | 34 |
| Maff2 family | 34 |
| Bacterial mobilization protein (MobC) family protein PcfF | 34 |
| DnaG type DNA primase | 34 |
| TraG/TraD family protein | 34 |
| Type IV secretory pathway C VirB4 components | 34 |
| DNA topoisomerase III | 34 |
| bacteriocin | 33 |
| Helix-turn-helix domain protein | 34 |
| Helix-turn-helix domain protein | 34 |
| Helix-turn-helix domain protein | 34 |
| Protein of unknown function (DUF3801) | 34 |
| Hypothetical protein | 34 |
| Hypothetical protein | 34 |
| Hypothetical protein | 34 |
| Hypothetical protein | 34 |
| Hypothetical protein | 34 |
| Hypothetical protein | 34 |
| Hypothetical protein | 34 |
| Hypothetical protein | 34 |

**Supplemental Table S1**
List of genes across the genome specific to *vanB* positive isolates in the study collection.
